# Molecular and histopathological identification of ovine neosporosis (*Neospora caninum*) in aborted ewes in Iraq

**DOI:** 10.14202/vetworld.2020.597-603

**Published:** 2020-03-30

**Authors:** Sattar J. J. Al-Shaeli, Ali M. Ethaeb, Hasanain A. J. Gharban

**Affiliations:** 1 Department of Anatomy and Histology, College of Veterinary Medicine, Wasit University, Wasit, Iraq; 2 Internal and Preventive Veterinary Medicine, College of Veterinary Medicine, Wasit University, Wasit, Iraq

**Keywords:** histopathology, Iraq, *Neospora caninum*, polymerase chain reaction, sheep

## Abstract

**Aim:**

The objective of the present study was to detect *Neospora caninum* DNA in the placenta of sheep and evaluate the association of risk factors to polymerase chain reaction (PCR) positive and histopathological analysis of the placenta and fetal tissue samples of aborted fetuses.

**Materials and Methods:**

Fresh placenta from 51 aborted ewes was collected for PCR assay. Placental and fetal tissues of aborted fetuses, including brain, heart, liver, lung, and thymus, were collected for histopathological analysis, besides the risk factor data were obtained during the time of sampling.

**Results:**

From 51 placentas examined by PCR, 13.73% appeared positive to *N. caninum* DNA. The relationship between PCR positive and the risk factors revealed a significant difference (p<0.05) in age of the dam, fetal age, feed source, water source, and the presence of other animals at farm, whereas the type of birth, stillbirth, and size of flock showed insignificant difference (p>0.05). Histopathological investigation of placental and fetal tissues of positive samples showed tissue cyst-like structure, necrotic foci, and infiltration of mononuclear cells. Other lesions were thickening in chorionic plate in placenta, severe vacuolization and death of neurons, microgliosis, demyelination, edema, and proliferation of astrocytes in brain. In addition, fibrous and fat deposition with stenosis in the heart, parenchymal necrosis, severe atrophy, vacuolization and hyalinization of hepatocytes, megakaryocyte, portal fibrosis in the liver, and interlobular septal thickening in lung without obvious lesions is seen in the thymus tissue samples.

**Conclusion:**

This is a unique study that confirmed *N. caninum* DNA in the placenta of aborted ewes in Iraq using PCR assay. Histopathological analysis of some aborted fetuses organs could provide a more confirmatory and reliable data for a significant role of neosporosis in increasing the rate of abortion in sheep, while the clinical data of risk factors could be used to control the transmission of *N. caninum* infection.

## Introduction

Neosporosis caused by *Neospora caninum* is one of the most important parasitic diseases which distributed globally among a variety of domesticated and wild animals resulting in high economic losses [1]. Historically, the parasite was first recognized in Norwegian dogs in 1984 and misdiagnosed as *Toxoplasma gondii*; however, the parasite is described as a new genus and new species in 1988 [2]. In ruminants, *N. caninum* is detected first in England in 1990 in congenitally infected lambs with the parasite tissue cysts in the brain and spinal cord [3]. The life cycle of the parasite, typified by three known infectious stages (tachyzoite, tissue cyst, and oocyst), involving mainly the ruminants and other ungulates as intermediate hosts and canids as definitive hosts [4]. Sheep may acquire a disease *in utero* by transplacental passage of tachyzoite from the dam to the fetus (vertical transmission) during gestation leading to miscarriage and birth of congenitally infected lambs, or postnatally by ingestion of sporulated oocysts found in contaminated foods or water (horizontal transmission) [5]. In cattle, *N. caninum* is considered the primary cause of abortion, although the clinical demonstration resembles ovine toxoplasmosis. Therefore, the significance of *Neospora* infection in sheep, including clinical, epidemiological, and economic still unrevealed [5,6]. However, limited studies showed potential role of *N. caninum* in sheep reproductive failure [7,8].

Today, many diagnostic techniques are available commercially for the detection of ovine neosporosis involving classical and developing methods. Recently, the histopathological screening of fetal tissues is the fundamental method used to determine the ovine abortion caused by protozoa [9]. Nevertheless, this single method is not precise because an inability to recognize infection with *N. caninum* and/or *T. gondii* due to morphological and lesions induced similarity, and thus, inaccurate diagnosis could be occurred [10]. This technical method limitation is supported by precise and sensitive molecular method that detected specific parasite DNA. Polymerase chain reaction (PCR) assay is accurate and widely used molecular techniques that identify specific DNA in limited samples [11].

Currently, the diagnosis of *N. caninum* as the leading cause of ovine abortion in Iraq is not well studied. Hence, this study is unique as the aim was to determine the association of *N. caninum* in flocks with significant abortions using the molecular PCR assay, as well as histopathological inspection of maternal placenta and fetus tissues, including brain, heart, liver, lung, and thymus, in addition to collected clinical data to identify the association between the risk factors and PCR-positive.

## Materials and Methods

### Ethical approval

The sample and data collection were approved by the Veterinary Medicine, Scientific and Ethical Committee of Wasit University.

### Study period and study animals

This study carried out during May 2017-January 2019 on private farms located in different rural areas, Wasit Province, Iraq. The owners had observed a dramatic increase in abortion cases approximately after the 3^rd^ month of pregnancy. Case history data reported that the affected flocks were immunized previously with brucellosis vaccine, treated with parasiticide (Ivermectin – 1% S/C and drenched orally). Besides, most aborted ewes were received two doses of oxytetracycline 20% (72 h interval) as preventive therapy before the season of mating.

Data regarding the risk factors concerned to study fetuses, their dams, and management were recorded during this study. The data obtained from the owners were included age of dam, type of birth, fetal age, stillbirth, size of flock, source of feeding, source of water, and presence of other animals at the farm.

### Sample collection

Under aseptic conditions, a total of 51 aborted ewes were subjected to a collection of fresh placentas in addition to aborted fetal tissues, including brain, heart, liver, lung, and thymus. From each placental sample, two biopsies were sectioned and kept into plastic containers, the first put primarily within the liquid nitrogen, and the other, as well as fetal tissue samples, were preserved within 10% buffered formalin. At the laboratory, the frozen placenta biopsies were kept in deep freeze (−80°C) till required for molecular assay. During the first 48 h of tissue buffering, 10% buffered formalin of all fixed biopsies was replaced every 12 h.

### Laboratory examination methods

#### Molecular examination

DNA extraction and purification

Based on the manufacturer’s instruction of the AccuPrep Genomic DNA Extraction Kit (BIONEER, South Korea), protocol V-3 was used to extract the DNA from frozen placental biopsies. Briefly, the samples were sliced, grind, and homogenized completely within a jar. A 25 mg of homogenized tissues were added into the Eppendorf with 200 ml of Buffer TL, 20 μl Proteinase K, and 5 μl RNase solutions then vortexed and incubated at 60°C for 1 h. A 200 μl of Buffer GC was added and incubated for 10 min at 60°C followed by adding 200 μl of absolute ethanol, vortexed, and transferred into the spin column that centrifuged at 13,000 rpm for 1 min. A 200 μl of absolute ethanol was added into new Eppendorf contained 400 μl of supernatant and centrifuged (13,000 rpm/1 min). The mixture transferred into the spin column that centrifuged (13,000 rpm/1 min). The spin column washed with 700 μl of washing buffer (W1) that centrifuged at 13,000 rpm for1 min. Repeatedly, the spin column was washed with 700 μl of W2 that centrifuged at 13,000 rpm for1 min with discarding the flow through and then recentrifuged with discarding of the flow through. The spin column transferred into new Eppendorf, and 200 μl of elution buffer (EL) was added and incubated (37°C/1 min) and subsequently centrifuged (13,000 rpm/1 min). The concentration and purity of obtained DNA (180-200 μl) was quantified using the Nanodrop (Thermo Scientific, UK). The DNA samples were stored at −20°C until to be used.

PCR reaction and analysis

PCR protocol to identify the DNA of *N. caninum* was conducted using the primers described previously from the ITS1 region of ribosomal DNA (JB1:5΄AGGAGGAGAAGTCGTAAGG3΄ and JB2:5΄GAGCCAAGACATCCATTGC3΄), which provided by Bioneer company (South Korea) to amplify the 500 bp DNA fragment [12]. PCR master mix was prepared based on the PCR kit (Bioneer, South Korea) at 25 μl final volume. PCR reaction performed into thermocycler (Bio-Rad, USA) involving 1 cycle of initial denaturation (95°C/5 min), 30 cycles of denaturation (94°C/1 min), annealing (60°C/1 min), and extension (72°C/1 min), final extension (72°C/5 min), and maintenance at 4°C. Final products were transferred into TAE-1X buffer of 2% agarose gel and applied to electrophoresis (80 Ma/100 V/1 h). Furthermore, 100-1500 bp of DNA ladder (INtRON, South Korea) and 10 μg/ml of ethidium bromide (Biotech, Canada) were used. The amplicons were visualized with Ultraviolet Transilluminator (Biobase, China).

#### Histopathology

Preserved placental and fetal tissue biopsies were dehydrated in gradual concentration of ethanol, cleared in xylene, and infiltrated and embedded in paraffin, and 4-5 mm was sectioned from the block by microtome. All slides that contained tissue sections were stained with hematoxylin and eosin stain following the manufacturer’s instruction (SYRBIO, Syria), and the tissues examined under light microscope (MEIJI, Japan) at 40×.

### Statistical analysis

Microsoft Office Excel (2013) and SPSS (*v*^23^) programs (IBM Corporation, USA) were used to analyze the data. Chi-square test was performed to identify the significant between the PCR-positive *N. caninum* cases and negative and to evaluate the association of risk factors variables to PCR positive. Statistical differences between values were considered significant at p<0.05.

## Results

**Figure-1:**
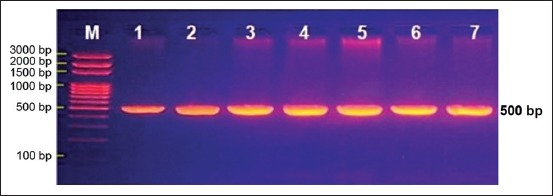
Gel electrophoresis of the conventional polymerase chain reaction of *Neospora caninum* DNA in aborted fetus placenta. Lane M: Ladder marker; lanes (1-7) represented positive samples.

Placenta of 51 aborted fetuses was examined by PCR for the detection of *N. caninum*. The result showed that out of 51 placental tissue samples, 7 (13.73%) samples were found positive (Figure-1), while 44 (86.27%) were negative.

Statistical analysis of the risk factors, including the type of birth, stillbirth, and size of the flock, did not show significant association with the positive PCR result. Whereas, the age of the dam, fetal age, feed source, water source, and presence of other animals at farm showed significant association (Table-1). The results were <4 years (20.83%), >3 months (18.75%), no stillbirth (14.89%), mixed (15.56%), river (18.75%), and presence of other animals at farm (14.58%).

**Table-1:** Relationship of risk factors to positive PCR results.

Factor	No.	PCR results	

		Positive (%)	Negative (%)
Age of dam			
≤4 years	24	5 (20.83)*	19 (79.17)
>4 years	27	2 (7.41)	25 (92.59)
Type of birth			
Single	36	5 (13.89)	31 (86.11)
Twin	15	2 (13.33)	13 (86.67)
Fetal age			
≤3 months	19	1 (5.26)	18 (94.74)
>3 months	32	6 (18.75)*	26 (81.25)
Stillbirth			
Yes	9	1 (11.11)	4 (100)
No	42	6 (14.29)	40 (85.11)
Size of flock			
≤100	13	2 (15.38)	11 (84.62)
>100-300	9	1 (11.11)	8 (88.89)
>300	29	4 (13.79)	25 (86.21)
Feed source (mostly)			
Pasture	5	0 (0)	5 (100)
Concentrate	1	0 (0)	1 (100)
Mixed	45	7 (15.56)*	38 (84.44)
Water source			
River	32	6 (18.75)*	26 (81.25)
Well	0	0 (0)	0 (0)
Mixed	15	1 (6.67)	14 (93.33)
Presence of other animals at farm			
Yes	48	7 (14.58)*	41 (85.42)
No	3	0 (0)	3 (100)

Significance

* (p<0.05)

### Histopathology

**Figure-2:**
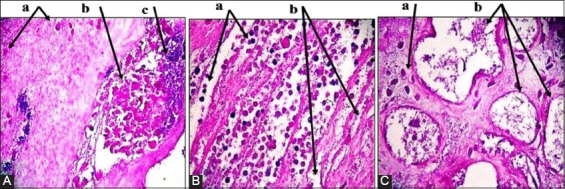
Histopathological findings of placental tissue samples. (A): (a) Thickened chorionic plate. (b) Mineralization within necrotic foci. (c) Tissue debris and cyst-like structure. (B): (a) Aggregation of multifocal polymorphic nuclear cells. (b) Necrosis in placental villi. (C): (a) MNCs infiltration. (b) Irregular cystic structure. Hematoxylin and Eosin was used to stain the tissue and images captured under the light microscope at 40×. The images are representative of the positive results.

**Figure-3:**
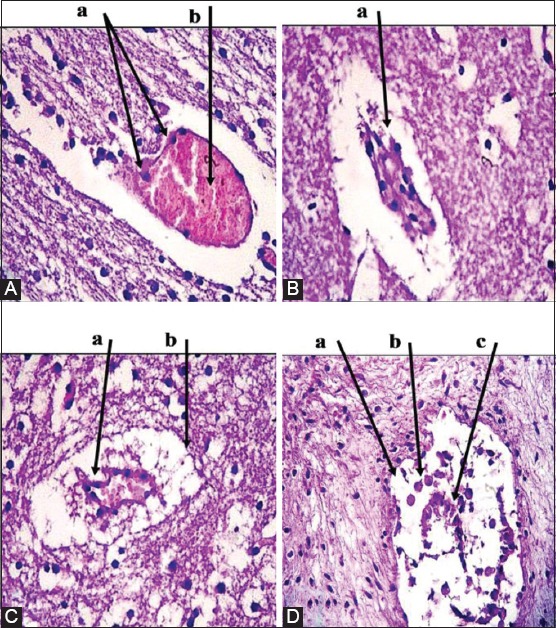
Histopathological findings of brain tissue samples. (A): (a) Microgliosis. (b) Congested blood vessels. (B): (a) Dead neurons and microglia cells. (C): (a) Mild aggregation of mononuclear cells. (b) Demyelination and edema. (D): (a) Irregular cystic cavity. (b) The proliferation of astrocytes. (c) Cellular debris. Hematoxylin and Eosin was used to stain the tissue and images captured under the light microscope at 40×. The images are representative of positive results.

**Figure-4:**
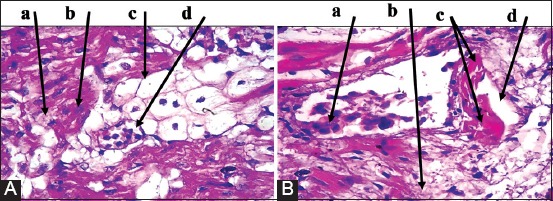
Histopathological findings of heart tissue samples. (A): (a) Fibrous deposition. (b) MNCs infiltration. (c) Steatosis. (d) Tissue cyst-like structure. (B): (a) MNCs infiltration. (b) Fibrous replacement of infarction area. (c) Necrotic muscle fiber. (d) Necrotic area. Hematoxylin and Eosin was used to stain the tissue which examined and images captured under the light microscope at 40×. The images are representative of the positive results.

**Figure-5:**
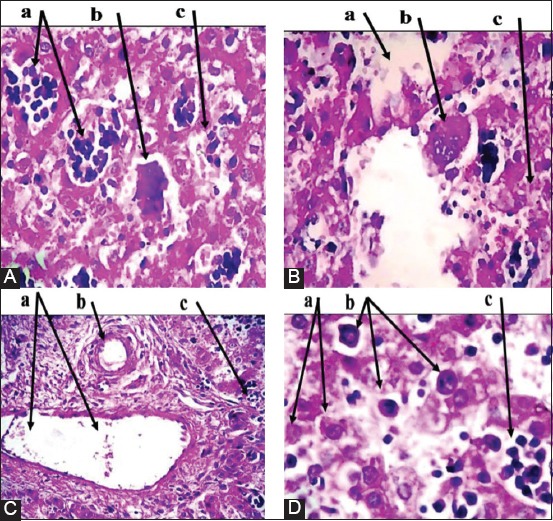
Histopathological findings of liver tissue samples. (A): (a) Tissue cyst-like structure. (b) Foreign body giant cells. (c) Mononuclear cells (MNCs) infiltration. (B): (a) Vacuolization and hyalinization of hepatocytes. (b) Megakaryocyte. (c) Parenchymal necrosis. (C): (a) MNCs aggregation in sinusoid. (b) Portal fibrosis associated with proliferation fibrous connective tissue. (c) Tissue cyst-like structure. (D): (a) Severe destruction of liver parenchyma. (b) Megakaryocyte. (c) Tissue cyst-like structure. Hematoxylin and Eosin was used to stain the tissue and images captured under the light microscope at 40×. The images are representative of the positive results.

**Figure-6:**
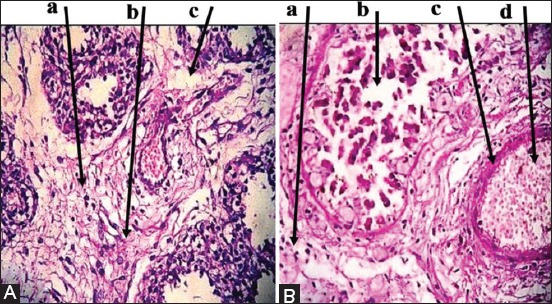
Histopathological findings of lung tissue samples. (A): (a) Thickening of intravalvular septa. (b) Mesenchymal cell proliferation. (c) Edema. (B): (a) Intensive necrosis of bronchial epithelial wall. (b) Large tissue cyst-like structures. (c) Thickening of the blood vessel. (d) Capillary congestion and dilation. Hematoxylin and Eosin was used to stain the tissue and images captured under the light microscope at 40×. The images are representative of the positive results.

Histopathological analysis of placental and fetal tissues showed several lesions within each organ as follows:

- Placenta: Multiple necrotic lesions with accumulation of tissue debris and tissue cyst-like structure, apparent thickening in chorionic plate, mineralization within necrotic foci (Figure-2A). Furthermore, aggregation of multifocal polymorphic nuclear cells, necrosis in placental villi (Figure-2B), with mononuclear cells (MNCs) infiltration, and irregular cystic structures (Figure-2C).
- Brain: Enlarged perivascular spaces, severe vacuolization of neuron, microgliosis, congested blood vessels (Figure-3A), with dead neurons and microglia cells (Figure-3B). In addition to sizeable intracellular vacuole with necrotic area, slight microglia proliferation, mild aggregation of MNCs, demyelination and edema (Figure-3C), as well as irregular cystic cavity, proliferation of astrocytes, and cellular debris (Figure-3D).
- Heart: Fibrous and fat deposition with slight infiltration of MNCs, steatosis, tissue cyst-like structure, and necrotic fibers (Figure-4A and B).
- Liver: Tissue cyst-like structure, parenchymal necrosis, severe atrophy (Figure-5A), with vacuolization and hyalinization of hepatocytes (Figure-5B). Furthermore, MNCs infiltration, particularly in sinusoids, multinucleated foreign body giant cells (FBGCs), portal fibrosis with proliferation of fibrous connective tissue (Figure-5C), with megakaryocyte, and severe destruction of liver parenchyma (Figure-5D).
- Lung: Interlobular septal thickening, mesenchymal cell proliferation, edema (Figure-6A), as well as tissue cyst-like structure, thickening of blood vessels, hyperplasia of bronchial epithelium, collagen deposition with capillary congestion, and dilation (Figure-6B).
- Thymus: No lesions.

## Discussion

*N. caninum* is an intracellular infectious disease that causes abortion or neonatal mortality in many animal species, particularly cattle, as well as sheep, goats, deer, and horses [13]. Neosporosis has not frequently identified as a cause of abortions in sheep, however, few cases in single animals or flocks have been reported [14,15]. In Iraq, the findings of the previous serological reports in sheep, goats, and cattle confirmed the possible infection of these animals by *N. caninum* [16,17]. Nonetheless, there are limited data related to the prevalence of *N. caninum* based on DNA detection in naturally infected sheep, and most information received is from experimentally and naturally infected cattle. The present study identified the precise prevalence of *N. caninum* in sheep in Iraq based on DNA screening (13.73%) which made this study unique. This finding identified the significant role of neosporosis in abortion in sheep and highlighted the importance of placenta as a source of infection. The result suggests that the aborted fetuses resulted from ovine neosporosis could be detected by molecular examination of placenta, which supported by the fact that the placenta is transported the infection to the fetal tissues [18]. It is clearly stated that the pathogenesis of neosporosis begins by transmitted the parasite through the placenta to the fetal tissues when these tissues undergo destruction concomitant with fetal and maternal immunity response destruction [19]. However, several other infections can cause flocks reproductive decline and abortion, which not included in this study. Detection of *N. caninum* DNA can be achieved from brain, heart, kidney, liver, and umbilical cord of aborted fetuses and blood of its dam rather than placenta as it is limited to obtain fresh placenta in specific time [8,11,17].

In the present study, the association of risk factors to PCR positive found significant differences in some results. It showed that the prevalence of ovine neosporosis increased in the dams with <4 years of age, and most positive fetuses aborted in the second trimester (pregnancy age of >3 months). This result could be due to high growth rate of the parasite, which transmitted to the fetus and induced abortion. Furthermore, the rate of endogenous transplacental infection may decrease in subsequent pregnancies due to the development of immunity [20]. It hypothesized that postnatal transmission could be prevented by developing immunity which achieved through early exposure of *Neospora* to young animals [21]. On the other hand, the neonates exhibited high levels of CD_14_^+^ monocytes, CD_80_ on the cell surface, and IL-1β which prevented *N. caninum* cellular invasion [22]. Concerning other risk factors, it identified that there were significant increases (p<0.05) in positive animals fed mostly on mixed, drank from rivers, and existed in contact with other animals at the same farm. As observed in many reports, the second way to spread the infection is consumption parasite oocysts or eggs contaminated food or water or grazing on contaminated pastures [23]. The serological result of the specific study showed that the higher anti-*N. caninum* antibodies were founded in cattle lived in farms with dogs in comparison with those without dogs, suggesting the active involvement of dogs in transmission of parasite to cattle [24]. The releases of oocysts of *N. caninum* through dog faces could contaminate the food and water which become the source of infection [25].

The evidence linking neonatal disease in sheep is based on findings of *N. caninum* organisms and associated antigens in tissue sections of naturally infected calves [3]. Histopathology is a standard screening method used for the detection of infection that caused abortion. In this study, the histopathological changes of PCR-positive placentas were varied from large MNCs foci with or without focal necrosis. The heavier infiltrate could trigger an intense adaptive immune response, leading to injury of the maternal placental junction and later endanger the fetus [26]. The screening of placentas exhibited mineralization which could not be related to *N. caninum* infection. In addition, increased thickness of chorionic plate is associated with a reduction in fetal and placental weight, as well as a diminished fetal and placental ratio (placental efficiency) at term [27].

Brain lesions frequently found in the cerebrum tissue of aborted fetuses, which suggested activation of inflammatory cells to initiate an immune reaction in response to the parasite. The various distributions of histopathological lesions and DNA of the parasite could be due to *Neospora* eradication [28]. It is clear that the perivascular spaces potentially having an immunological role, but more broadly a dispersive role for neural and blood-derived messenger [29]. Macrophages or microglia infiltration associated with nitric oxide synthase production were seen in mice brain that infected with *N. caninum* [30]. The released nitric oxide from active astrocytes and microglia caused neuron destruction through liberating glutamate by suppressing neuron respiration [31].

In heart, fibrous, and fat depositions with slight infiltration of MNCs, stenosis, *Neospora* like-structure, and necrotic fibers were the only seen in this study. Fibrous deposition might be a reactive state due to *N. caninum* infection or in response to an injury in heart muscle. The result of Lee *et al*. [32] showed that in mice, augmented fatty acid caused hypertrophy, wall thickness, and cardiac dysfunction due to the accumulation of fatty droplets. Stenosis or fatty change is an abnormal retention of fat within a cell or organ, which reflects an impairment of the normal process of synthesis and elimination of fat. Although stenosis can occur in the heart, the term is not specified and is assumed to refer to liver lipid metabolism [33,34]. A study of Dubey *et al*. [35] reported that the cause of death in aborted fetuses is the cardiac failure caused by *N. caninum*-associated myocarditis. In addition, numerous tachyzoites were detected in the extensive and severe myocardial lesions.

Lesions in the liver in response to protozoa or parasite cyst were consistent. Tissue cyst-like structures were seen among almost sections of the liver, and *N. caninum* bradyzoites were suspected to be seen within the lesions. In intermediate hosts, tachyzoites and tissue cysts are the infective stages. However, several studies refer to *N. caninum* as one of the suspected etiologic agents of hepatic lesions such as lymphocytic portal hepatitis, multifocal hepatocellular necrosis, focal hepatic necrosis, and fibrin thrombi in hepatic sinusoids [36,37]. The high number of tissue cyst-like structures concomitant with focal necrosis can be seen in the liver of aborted fetuses which suggested a high quantity of parasite that caused an acute infection due to epizootic form of dam primary infection. A limited number of parasites transmitted to fetuses from preexisting maternal infection under reactivation are likely the leading cause of sporadic abortion rather than primary infection [38].

In this study, pulmonary lesions were observed in PCR positive, in addition to interlobular septal thickening, which is uncommon as a predominant finding. Other lung lesions involved could be a sequelae of *N. caninum* infection as there were no data confirm that detected in this study.

## Conclusion

To date, limited data research has been conducted on the prevalence of *N. caninum* in Iraq. Therefore, the data of the present study is unique as it detected the prevalenceof *N. caninum* in aborted Iraqi ewes through quantifying DNA in placental tissues. Histopathological analysis of organs of aborted fetuses could provide more confirmatory and reliable data for a significant role of neosporosis in increasing the rate of abortions in sheep. Further studies are required to provide the essential data that may contribute to the detection of infection and their actual role in reproductive losses.

## Authors’ Contributions

SJJA and AME were responsible for histopathological analysis of placental and fetal tissue samples. HAJG was responsible for clinical and molecular examination. All authors participated in the drafting and revision of the manuscript.All authors read and approved the final manuscript.
